# The development and validation of tour guides internalized occupational stigma scale (TIOSS)

**DOI:** 10.1186/s12889-024-18519-5

**Published:** 2024-04-12

**Authors:** Zhiguang Fan, Xiaoli Shi, Li Liu, Shuhan Yang, Li Li

**Affiliations:** 1 Department of Psychology, School of Teacher Education, Shaoxing University, Shaoxing, 312000 People’s Republic of China; 2 School of Education, Jilin International Studies University, Changchun, 130117 People’s Republic of China; 3 School of International Cultural Tourism, Jilin International Studies University, Changchun, 130117 People’s Republic of China; 4 School of Economics and Management, Jilin Engineering Normal University, Changchun, 130052 People’s Republic of China

**Keywords:** Tour guides, Internalized occupational stigma, Reliability, Validity

## Abstract

**Background:**

Tour guides’ identification and internalization of occupational stigma may exacerbate their career development, perceived professional reputation and status, and mental health. The current study aimed to develop and verify the Tour guides Internalized Occupational Stigma Scale (TIOSS) to provide an effective tool for relevant quantitative research.

**Methods:**

The study developed an initial questionnaire through literature analysis, expert review, and semi-structured surveys. We conducted item analyses and exploratory factor analyses among 326 tour guides, and confirmatory factor analysis and reliability and validity tests among 315 tour guides.

**Results:**

The TIOSS consists of 21 items and is formed in three dimensions referring to Stigma Perception (SP), Status Loss (SL), and Career Denial (CD). The correlation coefficient values of the TIOSS total scale and dimension scores with the criterion instruments ranged from 0.17 to 0.68. In addition, the Cronbach’s α coefficients for the TIOSS and its dimensions ranged from 0.837 to 0.928, and the split-half reliability coefficients ranged from 0.843 to 0.916. The study also revealed that the TIOSS was consistent across genders.

**Conclusion:**

The TIOSS performed favorable reliability and validity to be a valid instrument to assess tour guides' internalized occupational stigma.

**Supplementary Information:**

The online version contains supplementary material available at 10.1186/s12889-024-18519-5.

## Introduction

Tour guides’ professional behaviors and attitudes are vital factors in influencing tourists’ travel satisfaction, evaluation of destination, consumption willingness, and purchase behavior [1]. Although it is undisputed that tour guides play a significant role in tourism, they are the ones most stigmatized among travel practitioners [2]. Recently, a news that tourists refused to buy bracelets and, then, their families were kicked off the bus by the tour guide went viral through media coverage. Most of the comments are filled with accusations and criticisms towards the entire tour guide profession. In addition, the news of tour guides' cheating, verbally abusive, threatening, coercing tourists, or changing destinations without authorization further fuels the stigma associated with the tour guides. Moreover, almost any profession is susceptible to being the object of stigma due to job content and attributes, while sordid work is particularly stigmatized [3]. Negative labels have been imposed on tour guides, such as “bad”, “sordid”, “dishonest”, and “less meaningful”, resulting in despising, disparagement, or even harassment towards them [2–4].

The intercourse between tour guides and tourists features a “servant relationship” [5]. The tour guides are responsible for not only explaining the local tourist attractions and cultural customs but also arranging transportation, accommodation, food, and shopping [6]. Meanwhile, throughout the tour, tour guides also necessitate working hard to meet various requirements, needs, and expectations of tourists to improve their satisfaction for pay rise [7]. In addition, negative reports of tour guides, which are widely disseminated in the news and social media, further aggravate the occupational stigma [8]. Currently, it is not uncommon to grasp unfavorable reports of tour guides. In particular, some tour guides who violated professional ethics or engaged in forced, abusive, and deceptive activities have seriously damaged their professional image [9]. It is asserted in a qualitative study that the occupational stigma of tourists towards tour guides, mainly embodied in morals, is the direct cause of triggering emotional reactions of suspicion, loathe, and contempt as well as criticism and indifference behavior [10].

Tour guides' internalized occupational stigma can be defined as the negative consequences of guides’ identification with dismissive occupation-related labels, perceptions of their occupation as groveling, immoral, and low prestige, resulting in perceived self-denial, discrimination, and rejection [11]. Individual’s internalized occupational stigma can lead to impaired occupational reputation and diminished sense of career value, declining social status and reputation, and threats to job happiness and mental health [12–16]. Moreover, internalized occupational stigma also harms the professional behaviors of practitioners. For instance, internalized occupational stigma significantly positively correlates with uncivilized behaviors, organizational deviance and disapproval, and turnover intention in the workplace [17–21]. Of particular concern is that the tour guide majored undergraduates elicit negative evaluations, such as unstable income and low respect, towards tour guides [22]. Stigmatization of their occupation can affect the career choice of college students and diminish their willingness to engage in related work, which further exacerbates the dilemma of the lack of tour guide professionals [23].

In the quantitative research on the occupational stigma of tour guides, the most commonly used tools are Stigma Consciousness Questionnaire (SCQ) [24] and Self-Stigma Scale (SSS) [25]. SCQ was originally used to measure the perceived stigmatized public perception of their work among call center workers [24]. Subsequently, the Occupational Stigma Consciousness Scale (OSCS) items, based on SCQ, were modified by researchers according to their research needs to assess stigma awareness among practitioners in different occupations [26]. Furthermore, Mak and Cheung developed the SSS, including the 39-item SSS-L and 9-item SSS-S versions [25]. Both the long and the short versions include Cognitive, Affective, and Behavioral dimensions. Moreover, the development of SSS was originally aimed for concealable minorities represented by mental health consumers, immigrants, and sexual minorities.

Hitherto, the SCQ and the SSS have been widely used to assess stigma awareness in diverse occupational populations. However, different occupations exhibit major diversity in the aspects of professional prestige, social status, work content, service targets, and job characteristics [27]. Practitioners in various industries differ in experiencing internalized occupational stigma. Since the SCQ and the SSS are not originally developed for tour guides, it is difficult to measure the stigmatized cognitive, emotional, and behavioral responses of tour guides to their profession. Additionally, the SCQ and the SSS measure stigma awareness among practitioners of a given occupation, rather than specifically for the internalized occupational stigma.

Therefore, the current study aimed to develop a tour guide internalized occupational stigma scale with satisfactory reliability and validity. This study employed conceptualizing stigma theory and self-verification theory as theoretical frameworks. As to social cognition, Link elaborated on the formation process of stigma and its negative impact and presented a Conceptualizing Stigma Model. It is posited from the theory that power disparities are a prerequisite for stigma formation, although discrepancies in the amount of power wielded by different groups in politics, economy, and society [28]. The stigma is developed through four steps. (a) distinguish and label between-group disparities; (b) associate group discrepancies with undesirable traits to constitute negative stereotypes by mainstream culture; (c) the emergence of social separation; (d) stigmatized people experience status loss, discrimination, and exclusion, being put in socially disadvantaged positions [3, 28].

The conceptualizing model focuses on the formation of public stigma and its detriments. By stark contrary, self-verification theory can better demonstrate how tour guides internalize public stigma into the self-occupational stigma. The theory suggests that people concentrate on and seek for self-discrepant feedback to enhance their sense of control and predictability of the real world [29]. When individuals receive feedback that disconfirms their self-conceptions, they maintain the homogeneity of their self-concept by creating a self-confirmatory social environment and by subjectively distorting reality information [30]. Individuals continuously receive and integrate external information, which further impacts the development of self-concept [31]. Individuals with a negative self-orientation are highly attentive to stigmatizing information and are more intensely affected by it. When tour guides perceive negative comments from tourists and the public, they are inclined to internalize and instill them into their self-concept, which can lead to the internalized occupational stigma.

According to the Conceptualization Stigma Model and self-verification theory, it is acknowledged that the public identifies and marks group differences based on the professional behaviors and job attributes of tour guides and assumes that all tour guides share similar profiles. The development of self-concept can be influenced when guides perceive and identify with stigmatizing evaluations from tourists and the public. Meanwhile, internalizing occupational stigma then provokes a sense of social isolation, perceived status loss, discrimination, and exclusion. Hence, internalized occupational stigma stems from the perception, identification, and application of public stigma information by tour guides. In the wake of the above theories, this study involved the tour guides' perceived stigma message, as well as the impact of internalized occupational stigma on professional status, mental health, and behavior.

The selection of the criterion tools was mainly based on social identity theory. This theory asserts that individuals assign themselves to specific groups through social categorization and obtain a positive social identity by social comparison between in-groups and out-groups [32]. Occupational identity is one of the important foundations of social classification. Positive social identification is obtained when the ingroup to which an individual belongs is superior to the outgroup in some aspects [33]. By contrast, if individuals fail to obtain affirmative or positive evaluations when comparing themselves to the outgroup, this may lead to a social identity threat [34]. Moreover, social identity threat can be classified into four categories: category threat, threat to group value, acceptance or prototypicality threat, and distinctiveness threat [35]. In this regard, a threat to group value refers to the identity threat caused by an individual’s belonging to a group that is disadvantageous when compared with an outgroup, or ingroup that is demeaned and undervalued.

Consequently, social identity threats may affect practitioners’ perceptions, attitudes, and behaviors towards their occupation and cause negative ramifications. When tour guides with high internalized occupational stigma perceive that their profession is being stigmatized and discriminated against, they may aware the social identity threats and affect their perception and evaluation of their careers. Previous studies have found that social identity threats are positively associated with low job engagement, low career commitment, low sense of value, psychological alienation, low self-esteem, perceived discrimination, and willingness to resign [36–39]. Therefore, tour guides who are affected by the threat of identity and have a high degree of internalized occupational stigma may maintain a lower level of identification and commitment to their profession, reduce their motivation to work, fail to gain professional improvement, and intend to resign. Accordingly, this study selected the Thriving at Work Scale (TWS), the Utrecht Work Engagement Scale (UWES-9), the Stigma Consciousness Questionnaire (SCQ), the Career Commitment Scale (CCS), the Occupational Disidentification Scale (ODS), the Workplace Incivility Scale (WIS), and the Intent to Leave Scale (ILS) as criterion instruments to examine the criterion validity of the Tour guides Internalized Occupational Stigma Scale (TIOSS).

In conclusion, the study aimed to develop the Tour guides Internalized Occupational Stigma Scale (TIOSS) to provide an effective tool for measuring tour guides internalized stigma. The study hypothesized that TIOSS shows satisfactory reliability and validity. Meanwhile, according to social identity theory, the study proposed that TIOSS was significantly positively correlated with SCQ, ODS, ILS, and UWES-9, and significantly negatively correlated with CCS, TWS, and WIS.

## Methods

### Item generation

The development of the questionnaire items consisted of 3 main aspects. First, a comprehensive literature review in the field of occupational stigma was conducted to sort out the relevant scales as a reference. The current study revised some items from scales, including the Stigma Consciousness Questionnaire (SCQ) [24], the Self-Stigma Scale (SSS) [25], the Brief Internalized Sex-work Stigma Scale [40], the Internalized Stigma of Mental Illness Scale (ISMI-10) [41], and Physician Internalized Occupational Stigma Scale (PIOSS) [42]. For instance, we revised the item 11 of the PIOSS from “I regret becoming a physician.” to “I regret being a tour guide.” A total of 8 questionnaire items were generated.

Second, one professor, one lecturer in tourism management, and three tour guides were invited to fully discuss the current situation of the internalized occupational stigma of tour guides and the potential detriments thereof. In the discussion, no clear rules or speaking orders were set, and the correctness or incorrectness of the discussion was not evaluated, and participants were encouraged to express their viewpoints as fully and truly as possible. This stage aimed to uncover as much as possible the characteristics of the internalized occupational stigma of tour guides to fill the gaps occured in the existing literature. For instance, experts analyzed the impact of tour guides’ forced shopping and threatening behavior in low-cost tours and zero-fare tours on the stigma of the entire profession. Furthermore, they also analyzed the impact of news media coverage on stigma, as well as the negative image of tour guides. Subsequently, the results of the discussion were compiled and analyzed by two graduate students in tourism management, and questionnaire items were developed. A total of 19 items were produced at this stage.

Third, 17 guides were selected for a semi-structured questionnaire survey. Five of them were male and 12 were female. The concept of occupational stigma and internalized occupational stigma was introduced to the respondents in detail before starting the survey. They were asked to complete the questionnaire based on their real experiences and feelings. Examples of questionnaire items: “How do you evaluate your own profession?” “In your opinion, what is the occupational stigma that the public holds about tour guides?” “What occupational stigmas have you ever encountered?” “How do you view the occupational stigma of tour guides?” “What negative effects do you think occupational stigma has on the tour guide industry?” “In your opinion, what negative effects might the internalized occupational stigma of tour guides have on themselves?”

At the end of the survey, the results were analyzed by 1 Ph.D. psychologist and 2 M.A. to distill the typical vocabulary related to the internalized occupational stigma of tour guides. The typical words extracted included: cheating, kickback, low level, forced shopping, induced/forced consumption, sales pitch, false propaganda, lack of respect, lack of trust, low social status, harassment, verbal abuse, defensive, ridicule, low income, unstable income, humble, helpless, aggrieved, angry, humiliated, low self-esteem, non-supported by family, limited personal development, and career transition. Based on the results of the semi-structured questionnaire, 28 items were discussed by a professor of tourism management and an associate professor of psychology, and compiled.

The 55 items arising from the above 3 sessions were aggregated. One associate professor of psychology and 1 Ph.D. in psychology evaluated each item individually. Items with repetitive content, ambiguity, low relevance, similar meaning, and lack of representativeness were removed. At the same time, the expressions of some items, and the vocabulary used, were modified to improve the conciseness, clarity, and comprehensibility of the questionnaire. After deletion, merging, and modification, a total of 36 items were retained.

### Pre-test and expert feedback

Three graduate students in tourism management with experience working as tour guides and 14 undergraduate students in tourism management were invited to perform the pretest. After completing the questionnaire, respondents were asked to discuss the comprehensibility of each item, the readability, the clarity of the questions, and the response burden. Among them, it was found that two of the items were leading, two were ambiguous, and four were difficult to read. Based on the feedback, the contents of the items were revised again, 4 items were deleted, and the presentation of the 4 items was improved. After Pre-test, 31 items were retained.

The initial scale of 31 items was sent to one psychology professor (with experience in scale development), two lecturers in tourism management, and three guides with extensive work experience for expert feedback. Experts were invited to evaluate the quality of the items, the readability, the linguistic expression habits, and the extent to which the items responded to the conceptual construct. Based on the results of the feedback, some of the phrases were fine-tuned (see Additional file 1). For example, the phrase “I find it difficult to find my value in my work” was changed to “I find it difficult to get a sense of value from my work”.

### Participants

This study was conducted in two phases of a questionnaire survey, and the respondents were all tour guides. Ten times the number of items was used as a criterion for calculating the minimum sample size [43]. The data in the first stage (Sample 1) were mainly used for item analysis and exploratory factor analysis. A total of 347 questionnaires were returned in the first stage, of which 326 were valid, with an effective rate of 93.95%. Moreover, data from the second stage (Sample 2) were mainly used for confirmatory factor analysis and reliability and validity testing. A total of 339 questionnaires were returned in the second stage, of which 315 were valid, with an effective rate of 92.92%. The demographic information is shown in Table 1.

**Table 1:** Socio-demographic characteristics

Category	Sample 1 (*n* = 326)		Sample 2 (*n* = 315)	
	N	%	N	%
Gender				
Male	112	34.36	118	37.46
Female	214	65.64	197	62.54
Age (Mean, SD)	33.95	(8.73)	35.46	(8.26)
Years of experience (Mean, SD)	9.29	(7.12)	10.31	(7.70)
Ethnic group				
Ethnic Han	310	95.09	298	94.60
Minority	16	4.91	17	5.40
Education				
High school (secondary vocational) and below	29	8.90	34	10.79
College	152	46.63	131	41.59
Undergraduate	119	36.50	130	41.27
Graduate student	26	7.98	20	6.35
Marital status				
Unmarried	117	35.89	95	30.16
Married	196	60.12	203	64.44
Divorced	11	3.37	14	4.44
Widowed	2	0.61	3	0.95
Job title				
Junior tour guides	265	81.29	213	67.62
Intermediate tour guide	44	13.50	71	22.54
Senior tour guide	17	5.21	31	9.84

## Instruments

### Tour guides internalized occupational stigma scale (TIOSS)

The initial TIOSS consisted of 31 items and the formal scale comprised 21 items. The scale adopted a Likert 5 rating scale 1 = strongly disagree, 5 = strongly agree), and the guides judged the degree of agreement with each item based on their true thoughts and attitudes. Higher scores indicate a higher level of internalized occupational stigma among the guides.

### Thriving at work scale (TWS)

TWS consists of 10 items divided into two dimensions of learning and vitality [44]. The scale is scored on a 7-point scale (1 = strongly disagree, 7 = strongly agree), with item 4 and item 8 being reverse scored. It is noted that higher scores indicating that the individual is more energetic at work and feels motivated to grow. The Cronbach’s α score for TWS in this study was 0.924.

### Utrecht work engagement scale (UWES-9)

UWES-9 consists of 9 items divided into three dimensions, including Vigor, Dedication, and Absorption [45]. The scale is scored on a 7-point scale (0 = Never, 6 = Always), and higher scores indicating higher levels of individual work engagement. The Cronbach’s α score for the UWES-9 in this study was 0.950.

### Stigma consciousness questionnaire (SCQ)

SCQ is composed of 10 items with a unidimensional structure [24]. The scale is scored on a 7-point scale (0 = strongly disagree, 6 = strongly agree) with higher scores indicating higher levels of perceived occupational stigma for the individual. The Cronbach’s α score for the SCQ in this study was 0.89.

### Career commitment scale (CCS)

CCS involves 8 items in a unidimensional structure [46]. The scale is scored on a 5-point scale (1 = strongly disagree, 5 = strongly agree), with reverse scoring for items 1, 3, and 7. Its higher scores suggest a higher level of commitment to the individual’s career. The Cronbach’s α score for the scale in this study was 0.775.

### Occupational disidentification scale (ODS)

ODS is composed of 3 items with a unidimensional structure [47]. The scale is rated on a 7-point scale (1 = strongly disagree, 7 = strongly agree). The higher score indicates that the tour guide identifies less with his or her profession. The Cronbach’s α score for the scale in this study was 0.768.

### Workplace incivility scale (WIS)

WIS consists of 7 items and is unidimensional in structure [48]. The scale uses a 5-point scale (0 = never, 4 = most of the time). The higher scores indicate that individuals experience disrespect, rudeness, or offense from superiors or co-workers in the workplace more frequently. The Cronbach’s α score for the scale in this study was 0.953.

### Intent to leave scale (ILS)

ILS consists of 4 items with a one-dimensional structure [49]. The scale is scored on a 5-point scale (1 = strongly disagree, 5 = strongly agree), with item 4 being reverse scored. And higher scores suggest that individuals are more likely to leave their jobs. The Cronbach’s α coefficient for the scale in this study was 0.754.

### Procedure

The survey was mainly completed in two ways. First, the questionnaires were distributed through the tour guide chapters of local tourism associations. The web survey link was sent to the tour guides after communicating with and obtaining consent from the association leaders in each province or city. Second, the data was collected through a snowball sampling method. The survey link was distributed to the guides in different regions by a guide chapter president. After the tour guides completed the questionnaire, they were asked to recommend potential subjects who had not participated in similar surveys and met the target profile of the study. And so on, it was gradually expanding the sampling range.

Informed consent was obtained from the subjects for this study. Before starting the survey, respondents were required to read the informed consent form carefully to understand in detail the anonymity, purpose, process, steps for completing the questionnaire, precautions, and confidentiality of the study. Only after the respondents checked the button “I have read the informed consent form in detail and voluntarily participate in this survey” could they begin to answer. After the data were collected, the researcher had to check the quality of the data and eliminate invalid questionnaires according to the following criteria. (a) all selected options; (b) identical in both positive and negative options; (c) response time greater than 2 standard deviations, or less than 2 standard deviations. The study was approved by the Ethics Committee of Jilin International Studies University (No. JY202104012).

### Data analysis

Item analysis and exploratory factor analysis (EFA) were performed on the data from Sample 1. In EFA, this study adopted the PAF method for factor extraction [50] and used the Promax method for oblique rotation [51] to determine the number of factors with eigenvalues greater than one [52]. Furthermore, to improve the stringency and simplicity of the factor analysis results, scale items were removed based on the following four criteria [43]: (a) factor loading values less than 0.40; (b) the existence of multiple loadings when the loading value of an item on two or more factors all exceeds 0.40; (c) commonality less than 0.30; (d) items lack of theoretical connection with the factor where it is located.

The data from sample 2 were used for confirmatory factor analysis (CFA) and reliability and validity tests. In CFA, the following recommendations were adhered for judging an ideal model fit: χ2/df < 3, RMSEA < 0.08, CFI, IFI, TLI > 0.90, PNFI, PCFI > 0.50 [53]. Pearson’s correlation analysis was used to calculate the correlation between the TIOSS and the criterion instruments. In addition, Cronbach’s α coefficient and split-half reliability were used as a method to assess reliability [54]. If the reliability coefficient is greater than 0.70, it indicates a high reliability of the scale [54].

This study further examined whether the TIOSS has cross-gender consistency. In the study, four models were developed, and the cross-group consistency was judged by comparing the difference in CFI and RMSEA between the models. If ∆CFI and ∆RMSEA were less than 0.01, the scales were indicated to be equivalent between males and females [55].

## Results

### Item analysis

The results showed (see Table 2) that the scores of the high group were significantly higher than those of the low group (*p* < 0.01). The correlation analysis results revealed that the correlation coefficient value between item 29 and the total score was 0.26, which was lower than the standard of 0.40. The correlation coefficients of the other items with the total score ranged from 0.47 to 0.78, which was higher than the criterion of 0.40. The Cronbach’s α value of the initial questionnaire was calculated to be 0.952. After deleting item 29, Cronbach’s α value increased and became 0.953.

**Table 2:** The results of the item analysis of the initial questionnaire

Item	Low score group (*N* = 88)		High score group (*N* = 88)		t-value	Corrected Item-Total Correlation	Cronbach’s Alpha If Item Deleted
	M	SD	M	SD			
1	1.90	1.02	3.94	1.12	12.69^**^	0.60^**^	0.951
2	1.64	0.85	3.93	1.12	15.32^**^	0.65^**^	0.951
3	2.98	1.20	4.58	0.69	10.84^**^	0.57^**^	0.951
4	1.73	0.87	3.97	1.01	15.77^**^	0.71^**^	0.950
5	2.07	1.09	4.52	0.77	17.22^**^	0.68^**^	0.951
6	1.81	1.03	4.23	0.96	16.19^**^	0.71^**^	0.950
7	1.26	0.54	2.89	1.25	11.25^**^	0.59^**^	0.951
8	2.18	1.28	4.27	0.75	13.19^**^	0.63^**^	0.951
9	1.85	1.00	4.25	0.79	17.63^**^	0.74^**^	0.950
10	1.95	1.01	4.14	0.85	15.58^**^	0.71^**^	0.950
11	2.59	1.12	4.70	0.51	16.12^**^	0.72^**^	.0.950
12	1.66	0.82	3.31	1.26	10.29^**^	0.58^**^	0.951
13	2.26	1.13	4.64	0.65	17.12^**^	0.73^**^	0.950
14	2.81	1.12	4.68	0.52	14.24^**^	0.69^**^	0.951
15	1.91	0.91	3.68	1.19	11.13^**^	0.61^**^	0.951
16	1.93	0.94	3.70	1.16	11.14^**^	0.61^**^	0.951
17	2.63	1.35	4.74	0.47	13.88^**^	0.69^**^	0.950
18	1.83	0.85	4.02	1.03	15.44^**^	0.70^**^	0.950
19	1.93	0.99	4.48	0.76	19.13^**^	0.76^**^	0.950
20	2.91	1.27	4.66	0.59	11.78^**^	0.64^**^	0.951
21	1.55	0.74	2.77	1.36	7.42^**^	0.47^**^	0.952
22	1.72	0.86	3.94	1.03	15.57^**^	0.69^**^	0.950
23	1.52	0.68	3.47	1.31	12.34^**^	0.66^**^	0.951
24	2.13	1.17	4.17	1.17	11.60^**^	0.60^**^	0.951
25	2.14	1.07	4.63	0.61	18.88^**^	0.78^**^	0.950
26	2.01	0.94	3.91	1.10	12.30^**^	0.64^**^	0.951
27	1.61	0.88	2.92	1.28	7.90^**^	0.53^**^	0.952
28	1.77	0.78	4.26	0.89	19.68^**^	0.75^**^	0.950
29	3.94	1.24	4.60	0.65	4.40^**^	0.26^**^	0.953
30	2.42	1.23	4.45	0.79	13.08^**^	0.64^**^	0.951
31	2.15	1.23	3.84	1.35	8.72^**^	0.51^**^	0.952

^**^p < 0.01

*M* Mean, *SD* Standard Deviation

### Exploratory factor analysis (EFA)

The Kaiser-Meyer-Olkin (KMO) index was 0.950, and the *p*-value of Bartlett’s chi-square was less than 0.001, indicating that the data were suitable for exploratory factor analysis. The results of the EFA (see Table 3) show that item 26, item 30, item 1, item 4, there are factor loadings of less than 0.40; item 3, item 25, item 19, there is a phenomenon of multiple loads; item 31 has a problem of low commonality.

**Table 3:** Results of EFA (Sample 1, *n* = 326)

Item	Factor 1	Factor 2	Factor 3	Commonality
2. In the eyes of many, the attitude of the tour guides towards tourists is bad.	**0.760**	−0.063	0.086	0.591
22. In the eyes of many, tour guides may be verbally abusive to tourists.	**0.750**	0.070	−0.006	0.627
5. In the eyes of many, tour guides may force tourists to shop.	**0.710**	0.196	−0.092	0.636
13. In the eyes of many tour guides may force tourists to enroll in fee-based programs.	**0.566**	0.383	−0.102	0.644
28. In the eyes of many, tour guides may deceive tourists.	**0.532**	0.274	0.057	0.588
16. In the eyes of many, a tour guide’s professional competence is poor.	**0.522**	−0.013	0.213	0.420
17. I think that the public holds a negative perception of tour guides.	0.056	**0.798**	−0.073	0.635
14. I think tourists are wary of guides.	0.094	**0.790**	−0.105	0.638
20. I think tour guides are often misunderstood.	0.087	**0.766**	−0.138	0.569
11. I think the social status of tour guides is declining year by year.	−0.073	**0.765**	0.121	0.627
9. I think tour guides are not respected by the public.	0.130	**0.512**	0.218	0.557
8. I think the career threshold for tour guides is low.	0.098	**0.463**	0.160	0.401
7. I regret being a tour guide.	−0.012	−0.077	**0.820**	0.602
12. Being a tour guide is not the job I expected.	−0.071	−0.018	**0.810**	0.588
27. I would hide my work from others.	0.112	−0.182	**0.706**	0.462
23. I think a tour guide is not a decent job.	0.090	0.019	**0.703**	0.584
18. I have seriously considered resigning.	0.022	0.199	**0.624**	0.582
24. I am reluctant to have my children work as tour guides.	−0.269	0.365	**0.592**	0.504
10. I have seriously considered switching careers.	−0.036	0.284	**0.589**	0.574
21. I don’t feel up to being a tour guide.	0.349	−0.353	**0.572**	0.409
15. My family does not support me to work as a tour guide.	0.044	0.115	**0.566**	0.436

The bold part is the factor and factor loading value of the item

After deleting the above items one after the other, perform the EFA again. The results are shown (see Table 3). Three factors were found to have eigenvalues greater than 1, with a cumulative total explained variance of 61.80%. Moreover, the loadings of the items ranged from 0.463 to 0.820, and the commonality ranged from 0.401 to 0.638. Based on the content of the items included in each factor, and concerning the theoretical framework, the three factors were named. Factor 1 included 6 items named stigma perception (SP); Factor 2 included 6 items named status loss (SL); and Factor 3 included 9 items named career denial (CD).

### Confirmatory factor analysis (CFA)

Results showed that the three-factor model fitted well, with each fit index being χ2/df = 2.368, RMSEA = 0.066, CFI = 0.927, IFI = 0.927, TLI = 0.916, PNFI = 0.768, and PCFI = 0.808, respectively. Whether the three-factor model of tour guides internalized occupational stigma is optimal in comparison to the other factor models required further validation. First, given that all three dimensions measure the construct of internalized occupational stigma, all items were combined to form a single-factor model. Second, since internalized occupational stigma is formed from the perception of stigma that can lead to a series of negative consequences. For this reason, the study combined perceived stigma with status loss or career denial, respectively, to construct two-factor models. The two-factor model (a) combines perceived stigma with status loss and career denial as a separate dimension, while the two-factor model (b) merges perceived stigma with career denial and status loss as a separate dimension. In addition, considering that both status loss and career denial measure the negative consequences of internalized occupational stigma, they were integrated into one dimension to construct the two-factor model (c).

The one-factor model, the two-factor model (a), the two-factor model (b), and the two-factor model (c) were used as competing models to conduct the CFA in turn. Furthermore, the results indicated (see Table 4) that the one-factor model, the two-factor model (b), and the two-factor model (c) had poor model fit indices. In addition, the model fit index of the two-factor model (a) was worse than that of the three-factor model, although it met the statistical requirements. Therefore, after holistic consideration, the three-factor model was deemed to be a more stable and reliable model.

**Table 4:** CFA & Competitive model fit index

Competitive model	χ^2^/df	RMSEA	CFI	IFI	TLI	PNFI	PGFI
Three-factor model	2.368	0.066	0.927	0.927	0.916	0.768	0.808
One-factor model	4.531	0.106	0.808	0.810	0.783	0.680	0.716
Two-factor model(a)	2.644	0.072	0.911	0.912	0.899	0.762	0.803
Two-factor model(b)	4.160	0.100	0.829	0.831	0.806	0.694	0.731
Two-factor model(c)	4.001	0.098	0.838	0.839	0.816	0.702	0.738

*RMSEA* Root-Mean-Square Error Of Approximation, *CFI* Comparative Fit Index, *IFI* Incremental Fit Index, *TLI* Tucker-Lewis Index, *PNFI* Parsimony Normed Fit Index, *PGFI* Parsimony Goodness-of-Fit Index

### Criterion-related validity

As shown in Table 5, the correlation coefficients between the TIOSS, SP, SL, CD with each criterion tool is 0.17 to 0.68.

**Table 5:** Criterion-related validity analysis of TIOSS

	1	2	3	4	5	6	7	8	9	10	11
1.TIOSS	–										
2.SP	0.87^**^	–									
3.SL	0.85^**^	0.75^**^	–								
4.CD	0.86^**^	0.55^**^	0.55^**^	–							
5.TWS	−0.42^**^	−0.28^**^	−0.17^**^	−0.54^**^	–						
6.UWES-9	−0.41^**^	− 0.30^**^	− 0.23^**^	− 0.48^**^	0.61^**^	–					
7.SCQ	0.60^**^	0.56^**^	0.50^**^	0.50^**^	−0.13^**^	− 0.16^**^	–				
8.CCS	−0.54^**^	−0.31^**^	− 0.32^**^	− 0.67^**^	0.50^**^	0.43^**^	− 0.25^**^	–			
9.ODS	0.55^**^	0.44^**^	0.35^**^	0.58^**^	−0.40^**^	− 0.31^**^	0.50^**^	− 0.36^**^	–		
10.WIS	0.40^**^	0.29^**^	0.26^**^	0.45^**^	−0.36^**^	− 0.23^**^	0.29^**^	−0.29^**^	0.43^**^	–	
11.ILS	0.55^**^	0.34^**^	0.29^**^	0.68^**^	−0.48^**^	− 0.37^**^	0.27^**^	−0.69^**^	0.36^**^	0.28^**^	–
Mean	64.69	18.49	22.59	23.61	56.99	48.63	29.81	23.81	10.29	7.18	10.63
Standard Error	16.58	6.10	5.13	8.08	10.59	13.20	7.11	5.85	4.66	6.51	3.56

^*^p < 0.05, ^**^p < 0.01

*TIOSS* Tour guides Internalized Occupational Stigma Scale (TIOSS), *SP* Stigma Perception, *SL* Status Loss, *CD* Career Denial, *TWS* Thriving at Work Scale, *UWES-9* Utrecht Work Engagement Scale, *SCQ* Stigma Consciousness Questionnaire, *CCS* Career Commitment Scale, *ODS* Occupational Disidentification Scale, *WIS* Workplace Incivility Scale, *ILS* Intent to Leave Scale

### Reliability analysis

In the results (see Table 6), Cronbach’s α scores for the TIOSS and the SP, SL, and CD dimensions ranged from 0.837 to 0.928, and the split-half reliability coefficients ranged from 0.843 to 0.916.

**Table 6:** The reliability coefficient of TIOSS

	Cronbach’s α	Split-half reliability
TIOSS	0.928	0.916
Stigma Perception	0.878	0.889
Status Loss	0.837	0.843
Career Denial	0.883	0.905

*TIOSS* Tour guides Internalized Occupational Stigma Scale

### Cross-gender consistency analysis

The study first developed a Configural Invariance Model (M1), which means that there are no between-gender group constraints placed on the parameter estimates such as the same number and attribution of items. Second, a Weak Invariance Model (M2) was constructed, which is based on M1 to impose the between-gender group equality constraints on the factor loadings. Third, a Strong Invariance Model (M3) was constructed, which was based on M2 with equal intercepts. Forth, the Strict Invariance Model (M4), which was based on M3 to impose equality constraints on the disturbance variances. The results demonstrated (see Table 7) that the models of M1, M2, M3, and M4 fitted well and satisfied the prerequisites for conducting cross-group consistency tests. In the comparisons between M2 and M1, M3 and M2, and M4 and M3, both ∆CFI and ∆RMSEA were less than 0.01, indicating that TIOSS has cross-gender equivalence.

**Table 7:** Results of cross-gender equivalence analysis

Model	χ^2^/df	CFI	IFI	TLI	PNFI	PCFI	RMSEA (90%CI)	∆CFI	∆RMSEA
M1	1.739	0.920	0.921	0.908	0.726	0.802	0.049 (0.042 ~ 0.055)		
M2	1.730	0.917	0.918	0.909	0.755	0.839	0.048 (0.042 ~ 0.054)	−0.003	− 0.001
M3	1.755	0.910	0.910	0.906	0.784	0.877	0.049 (0.043 ~ 0.055)	−0.007	0.001
M4	1.724	0.908	0.908	0.910	0.823	0.928	0.048 (0.042 ~ 0.054)	− 0.002	−0.001

*M1* Configural Invariance Model, *M2* Weak Invariance Model, *M3* Strong Invariance Model, *M4* Strict Invariance Model, *CFI* Comparative Fit Index, *IFI* Incremental Fit Index, *TLI* Tucker-Lewis Index, *PNFI* Parsimony Normed Fit Index, *PGFI* Parsimony Goodness-Of-Fit Index, *RMSEA* Root-Mean-Square Error Of Approximation

In the analysis of gender differences in TIOSS total and dimension scores by independent sample t-test, it was found that men scored significantly higher than women in the TIOSS total (t = 2.66, *p* = 0.008), PS (t = 2.65, *p* = 0.008), and SL (t = 3.04, *p* = 0.003) dimensions, whereas in CD (t = 1.63, *p* = 0.105) dimension scores were not significantly different (see Table 8).

**Table 8:** Results of the variance analysis of the total score and each dimension of TIOSS

	Male(M ± SD)	Female (M ± SD)	t	*p*
TIOSS	68.00 ± 18.14	62.70 ± 15.28	2.66	0.008
Stigma Perception	19.65 ± 6.32	17.79 ± 5.86	2.65	0.008
Status Loss	23.71 ± 5.14	21.92 ± 5.02	3.04	0.003
Career Denial	24.64 ± 9.45	22.99 ± 7.10	1.63	0.105

*M* Mean, *SD* Standard Deviation

## Discussion

The current study reviewed the previous related literature and explored the concept of tour guides internalized occupational stigma. On that basis, the study strictly followed the scale development process to create scale items under the theoretical framework of the Conceptualizing Stigma Model (CSM) and self-validation theory. Afterward, the study surveyed 641 tour guides in two stages to test validity and reliability to finally form the 3-dimension, 21-item Tour guides Internalized Occupational Stigma Scale (TIOSS). It was concluded that TIOSS showed favorable construct validity and also high reliability as a valid tool to assess the internalized occupational stigma of tour guides.

The three dimensions of Stigma Perception (SP), Status Loss (SL), and Career Denial (CD) explored in this study have been previously described in the literature. As proposed in the Conceptualizing Stigma Model (CSM), stigma is composed of five constituents: labeling, stereotyping, separation, status loss, and discrimination [28]. Stigma perception is a guide’s awareness of negative labels and stereotypes associated with his or her profession, which is mainly reflected in the way guides treat tourists and behave. High scorers will obviously mean perceived strong negative evaluations from the public towards the tour guides, such as deception, poor attitude, coercion, and poor professional competence. Stigma perception reflects in the related components of labeling and stereotyping.

Loss of status and career denial represent the unfavorable consequences of stigma perception. Status loss echoes the guides’ perceptions of the social status and respect of their profession. High scorers are inclined to believe that tourists lack trust to and respect for tour guides and the mutual relations are antagonistic and guarded. This dimension incorporates both separation and status loss in the CSM. Career denial refers to the guides’ negative judgment of their career value and development. High scorers tend to deny their career choices and professional competence and try to reduce or eliminate the negative effects of stigma by hiding, resigning, and changing career. It is posited in the CSM that status loss is the originator of discrimination, which manifests itself in reduced life quality, declined income, unemployment, and decreased social interaction. The career denial dimension explored in this study is a concrete manifestation of discrimination in the career field.

The study further examined the validity of the TIOSS for explaining the validity of the scale and its predictive effect on actual behavior [56]. In previous studies, occupational stigma was observed to be negatively influencing the organizational atmosphere as well as practitioners’ work attitudes, behaviors, intergroup relations, professional identity, and psychological well-being [57]. The present study also reached consistent findings in its analysis of the criterion validity of internalized occupational stigma of tour guides. In addition, it was noted that TIOSS was significantly negatively related to the sense of job thriving, work engagement, and career commitment, and significantly positively related to occupational stigma consciousness, occupational disidentification, workplace incivility, and intention to resign. Notably, stigma originates from a wide range of sources, such as gender, race, organization, occupation, and country [58]. The formation of individual stigma is mainly based on inferences about the abilities and characteristics of the stigmatized population. In contrast, occupational stigma is not born because of the individuals' inherent attributes, but is formed based on the content of the job or the workplace [59]. This entails that once an individual is engaged in a job, a specific association is established between the negative labels and the occupation. Normally, as individuals depart from their jobs, the perceived occupational stigma is also detached from it. Thus, low occupational identity, low occupational commitment, and separation serve as means of coping with occupational stigma.

Consequently, if individuals accept, recognize, or even internalize occupational stigma, this will affect their development of self-concept. Even if the individual ceases to work in the related job, it can have an ongoing adverse impact on the self-development then and in the future [11]. To reduce the detrimental effects of occupational stigma, individuals may implement strategies to reconstruct their cognitive approach, such as lowering their judgments of occupational value and increasing their sensitivity to stigmatizing information [59, 60]. Therefore, individuals with high internalized occupational stigma are inclined to perceive their occupation as being disparaged by others, experience significant interpersonal stress, and lower occupational identity and career commitment, as well as to easily resign. In addition, according to social identity theory, people develop social identities according to their occupations and make specific connections between group characteristics and themselves [61]. Thus, the identity threat posed by stigma can render this alert to consume psychological resources, trigger avoidance and withdrawal behaviors, which subsequently leads to low work engagement and a lack of work dynamics and learning initiatives.

Whether there are gender differences in internalized stigma is an ever-focused issue for scholars [62, 63]. However, inconsistent conclusions have been drawn in studies of different types of occupational stigma [64–67]. The inequivalence of measurement instruments may be a potential explanatory reason. If the measurement instruments are unequal for different genders, the results of inter-group comparisons may be biased [68]. For this reason, this study further examined the consistency of TIOSS across gender. It was found that the configural invariance model, weak invariance model, strong invariance model, and strict invariance model all validated, meaning that the attribution, factor loading, intercept, and error variance of the items were the same. The results of the cross-gender consistency test indicated that TIOSS met the prerequisites for conducting comparisons of group differences.

Based on the above, the study further explored gender differences in internalized occupational stigma among tour guides. As the results demonstrated, men scored significantly higher than women on the total TIOSS score and the perceived stigma and status loss dimensions. In previous studies of public stigma, it was determined that men showed higher levels of stigma than women [69], which coincides with the study’s findings. Analysis of the causes may be due to the variation in societal role expectations for the different genders. Men tend to have stronger achievement motivation and higher expectations for their own career development [70]. This leads men to be more concerned about their social status, professional reputation, career development, and respectability of their profession, and to be more sensitive to stigmatizing information.

This study possesses certain values. The study compiled the TIOSS with satisfactory reliability and validity based on the strict adherence to the scale development process. This study is the first internalized occupational stigma scale developed for the tour guide profession, which provides a scientific and effective measurement tool for potential empirical studies. Reducing the stigmatization of tour guides and enhancing public understanding, respect, and trust in tour guides can facilitate the occupational identity of tour guides as well as promote career development. Furthermore, the TIOSS developed in this study can be used as an effective tool for identifying the internalized professional stigma of tour guides and for evaluating the effectiveness of intervention practices. This study explored and validated the intrinsic structure of the internalized occupational stigma of the tour guide to help enrich the connotation and better understand its psychological structure. This study not only verified that the Conceptualizing Stigma Model and self-verification theory are applicable in the field of occupational stigma, but also explored the three-factor structure as a useful supplement to the related theories. Moreover, the analysis of the criterion validity in this study is not only a test of scale validity but also an enrichment of social identity theory. From the perspective of individual occupational identity, the study indicated that social identity threat can not only bring about negative effects on individuals’ self-concept and psychological health but also affect career development.

### Limitations

Some limitations of this study require further exploration in ensuing studies. First, the study is plagued with the problem of the underrepresentation of subjects. The subjects of the current study were sourced from most provinces in China and were fairly representative. However, the study adopted convenience sampling and snowball sampling rather than strict random sampling. In prospective studies, it is necessary to adopt a strict sampling method and expand the sampling range to improve the representativeness of the sample. Second, stigma, as a typical cultural phenomenon, may have certain differences in different social backgrounds, economic conditions and cultures. More evidence is required to assess the internalized occupational stigma of tour guides in other countries. Furthermore, the study found that men had higher levels of internalized occupational stigma than women, and it is unclear whether this conclusion applies to other cultural backgrounds. Therefore, future studies can consider to test the reliability and validity of TIOSS in other cultural contexts, and further analyze whether there is a gender difference in internalized occupational stigma. Third, the study did not examine retest reliability, which makes it difficult to explain whether the scale is consistent and stable across time. Future studies examining the reliability-validity index of the TIOSS are necessary to better interpret the validity of the scale. Fourth, the predictive effect of the TIOSS on actual behavior still deserves further validation, especially the antecedent and outcome variables of internalized occupational stigma are in need of more systematic exploration. Fifth, the adoption of a self-report approach for assessing internalized occupational stigma in tour guides makes it difficult to avoid the negative effects of social desirability effects and response preferences. Therefore, in future research, there is also a need to develop different forms and contents of assessment by incorporating the latest psychometric theories and methods.

Apart from the above limitations, research on internalized occupational stigma among tour guides is still in its initial phase, and many issues remain to be further explored and addressed. For instance, although theoretical studies have provided a structural framework for the analysis of internalized occupational stigma, a large number of empirical studies are still needed to provide factual evidence to better explain the mechanisms of stigma formation. Moreover, and most importantly, the development of simple and accessible stigma interventions that can be applied to the tour guides population is of great significance for the stability of the tour guide workforce as well as for career development. Also, TIOSS can be employed for identifying priority intervention populations. For guides with intense internalized occupational stigma, they can employ adaptive strategies such as focusing on the positive values and strengths of the occupation as well as ignoring negative evaluations and disadvantages to reduce the detrimental effect of occupational stigma.

## Conclusions

The TIOSS developed in the study is composed of 21 items, which can be divided into 3 dimensions: stigma perception (SP), status loss (SL), and career denial (CD). The TIOSS features favorable reliability and validity and can be regarded as an effective tool for assessing tour guides’ internalized occupational stigma. In addition, it is necessary to scientifically identify and promptly intervene to improve the professional identity of tour guides, considering the significant ramification caused by occupational stigma to the tour guide profession.

### Supplementary Information

**Supplementary Material 1.**

## Data Availability

The datasets used or analyzed during the current study are available from the corresponding author upon reasonable request.

## Abbreviations

M1: Configural Invariance Model
M2: Weak Invariance Model
M3: Strong Invariance Model
M4: Strict Invariance Model
SP: Stigma Perception
SL: Status Loss
CD: Career Denial
CI: Confidence Interval
ILS: Intent to Leave Scale
SSS: Self-Stigma Scale
SCQ: Stigma Consciousness Questionnaire
WIS: Workplace Incivility Scale
ODS: Occupational Disidentification Scale
CCS: Career Commitment Scale
TWS: Thriving at Work Scale
CFI: Comparative Fit Index
IFI: Incremental Fit Index
GFI: Goodness-of-fit Index
TLI: Tucker-Lewis Index
CSM: Conceptualizing Stigma Model
EFA: Exploratory Factor Analysis
CFA: Confirmatory Factor Analysis
PNFI: Parsimony Normed Fit Index
PGFI: Parsimony Goodness-Of-Fit Index
TIOSS: Tour guides Internalized Occupational Stigma Scale
ISMI-10: Internalized Stigma of Mental Illness Scale
SRMR: Speech-to-reverberation Modulation Energy Ratio
RMSEA: Root-mean-square Error of Approximation
UWES-9: Utrecht Work Engagement Scale
OSCS: Occupational Stigma Consciousness Scale
