# Supplementary material for: The development and validation of tour guides internalized occupational stigma scale (TIOSS)
Source: BMC Public Health. 2024 Apr 12;24:1018. doi: 10.1186/s12889-024-18519-5 (PMC11010348; doi:10.1186/s12889-024-18519-5)
Supplement: Supplementary file 1 — Supplementary Material 1. [file 12889_2024_18519_MOESM1_ESM.pdf]

Tour guides Internalized Occupational Stigma Scale (TIOSS)

Any profession may be negatively evaluated by the public, the clients or the media, and tour guides are no exception. The following are some statements about the occupation of a tour guide, please judge them according to your true feelings and ideas. Where, 1 stands for "strongly disagree" and 5 stands for "strongly agree".

1. In the eyes of many, the attitude of the tour guides towards tourists is bad.
2. In the eyes of many, tour guides may force tourists to shop.
3. I regret being a tour guide.
4. I think the career threshold for tour guides is low.
5. I think tour guides are not respected by the public.
6. I have seriously considered switching careers.
7. I think the social status of tour guides is declining year by year.
8. Being a tour guide is not the job I expected.
9. In the eyes of many tour guides may force tourists to enroll in fee-based programs.
10. I think tourists are wary of guides.
11. My family does not support me to work as a tour guide
12. In the eyes of many, a tour guide's professional competence is poor.
13. I think that the public holds a negative perception of tour guides.
14. I have seriously considered resigning.
15. I think tour guides are often misunderstood.

16. I don't feel up to being a tour guide

17. In the eyes of many, tour guides may be verbally abusive to tourists.

18. I think a tour guide is not a decent job.

19. I am reluctant to have my children work as tour guides.

20. I would hide my work from others.

21. In the eyes of many, tour guides may deceive tourists.
